# Changes in the Expression of Smooth Muscle Cell–Related Genes in Human Dermal Sheath Cup Cells Associated with the Treatment Outcome of Autologous Cell–Based Therapy for Male and Female Pattern Hair Loss

**DOI:** 10.3390/ijms23137125

**Published:** 2022-06-27

**Authors:** Yuzo Yoshida, Miki Takahashi, Haruyo Yamanishi, Yosuke Nakazawa, Jiro Kishimoto, Manabu Ohyama

**Affiliations:** 1 Regenerative Medicine Research & Business Development Section, Shiseido FS Innovation Center, Yokohama 220-0011, Japan; miki.takahashi1@shiseido.com (M.T.); haruyo.yamanishi@shiseido.com (H.Y.); yosuke.nakazawa1@shiseido.com (Y.N.); jiro.kishimoto@hb.nagase.co.jp (J.K.); 2 Department of Dermatology, Kyorin University Faculty of Medicine, Tokyo 181-8611, Japan

**Keywords:** hair follicles, dermal sheath cup cells, androgenetic alopecia, cell-based therapy, biomarker, smooth muscle cells, SRF, CALD1

## Abstract

In a clinical study of autologous cell–based therapy using dermal sheath cup (DSC) cells, the treatment of hair loss showed improvements. However, the outcomes were variable. Here, correlations between marker gene expression in DSC cells and treatment outcomes were assessed to predict therapeutic efficacy. Overall, 32 DSC cell lines were used to evaluate correlations between marker gene expression and treatment outcomes. Correlations between vascular pericyte and preadipocyte marker expression and treatment outcomes were inconsistent. As smooth muscle cell markers, MYOCD correlated negatively with treatment outcomes and SRF consistently demonstrated an inverse correlation. Additionally, CALD1 correlated negatively and ACTA2 correlated inversely with treatment outcomes. DSC cell lines were divided into good and moderate/poor responders to further investigate the correlations. SRF and CALD1 were lower in a good responder compared with a moderate responder. Next, DSC cells were differentiated toward dermal papilla cells. Dermal papilla markers SOX2 and LEF1 before differentiation had moderate positive and inverse correlations with the treatment outcome, respectively. SOX2 after differentiation more consistently demonstrated a positive correlation. Significant downregulation of smooth muscle–related genes was also observed after differentiation. These findings revealed putative markers for preclinical evaluation of DSC cells to improve hair loss.

## 1. Introduction

Hair loss can greatly affect the quality of life of affected individuals. Thus, a great demand exists for its remedy. Male and female pattern hair loss is a common hair loss condition in which hair follicles miniaturize because of accelerated hair cycles, resulting in a decrease in hair density in defined anatomical locations, namely the frontal and vertex area of the scalp in males and the vertex with accentuation to the mid-frontal area sparing the frontal fringe in females [1,2,3,4,5].

Previous studies have demonstrated that the size of the dermal papilla (DP)—a highly specialized mesenchymal cell aggregate that plays a central role in hair follicle development and regeneration—is reduced in miniaturized hair follicles, which is characteristic of male pattern hair loss (androgenetic alopecia) [6]. Additionally, the number of DP cells in individual hair follicles correlates with their size [7], and loss of DP cells decreases the diameter of the hair shaft [8]. Accordingly, a hypothesis has been raised: in such forms of alopecia, disruption of the traffic balance between DP cells and their precursors during the hair cycle may lead to a DP size reduction and consequently to hair miniaturization [9]. If this theory is correct, enlargement of a once miniaturized hair shaft may be possible by supplementation of DP cells or their precursors in affected hair follicles.

The dermal sheath (DS) is a sheath-shaped connective tissue that encases the entire hair follicle; it is located outermost to the hair follicle and connects with the DP via a stalk-like structure at the bottom of the hair bulb. In the human hair follicle, the DS is formed from multiple connective tissues that are relatively thicker than those in mice, consisting of a thin layer of connective tissue [10,11]. The DS has hair follicle regenerative potential [12,13] (for review see [14]). The dermal sheath cup (DSC), a part of the DS at the bottom of the hair bulb region, is of particular interest because it contributes to hair follicle regeneration [12,15,16]. DSC cells can be harvested from the DSC by explant culture combined with microdissection [17,18]. When DSC cells are transplanted into a wounded ear, they migrate and incorporate into the DP and DSC of existing hair follicles to prolong the hair growth phase [17]. Furthermore, hair follicles generate ectopically by transplantation of DSC cells into the foot pad [17]. More importantly, we recently showed that injection of human DSC cells into a human reconstituted skin equivalent with hair follicles resulted in their incorporation within the DS, and notably the DSC, of hair follicles [19]. Moreover, a previous study directly demonstrated that a portion of DS cells is supplied to the DP structure during the hair cycle and that the number of DP cells derived from DS cells increases by repeating hair cycles [20]. These results eloquently indicate the potential of DSC cells for the treatment of male and female hair loss by reverting the miniaturized hair follicle phenotype by enlarging DPs.

On the basis of these observations, a proof-of-concept clinical study of autologous cell–based therapy using human DSC cells for male and female pattern hair loss has been carried out, demonstrating that the total hair density and cumulative hair diameter are indeed increased by transplanted DSC cells with statistical significance [21]. However, the outcomes of DSC cell transplantation were variable among the recipients, suggesting that the bioactivity of transplanted DSC cells is not equal (Supplementary Figure S1). This observation implies the necessity of assessing the hair-regenerative capacity of DSC cells prior to transplantation, which should provide a valuable tool to predict therapeutic outcomes. To date, methodology has not been established to evaluate such activity of DSC cells.

DS cells have been shown to act as myofibroblasts [22], cells with smooth muscle cell–like characteristics, and emerge at the time of wound healing [23]. The origin of myofibroblasts has been speculated to be DS cells or vascular pericytes based on their commonality of smooth muscle cell marker expression such as α-smooth muscle actin [24], encoded by the ACTA2 gene. Thus, it is reasonable to speculate that DSC cells may also have smooth muscle cell– and/or vascular pericyte–like properties. Considering their capacity for tissue regeneration, it is tempting to hypothesize that expression of important molecules in the determination of their fate or maintenance of intrinsic properties correlates with the DSC cell capacity to differentiate into DP cells and eventually restore miniaturized hair follicles. MYOCD and SRF are upstream regulators of smooth muscle cell differentiation [25,26]. The expression of pericyte marker genes PDGFRβ, CSPG4, and nestin can be assessed to evaluate vascular pericyte–like properties [27,28,29]. Additionally, PPARγ and CEBPα, upstream regulators governing preadipocyte differentiation to mature adipocytes [30], may reflect tissue regeneration potential.

In this study, the correlation between the magnitude of the aforementioned DSC cell properties with their characteristics to differentiate into DP cells and treatment outcomes was assessed to elucidate factors for predicting the efficacy of DSC cell transplantation prior to treatment. To enhance differentiation of DSC cells to DP cells in vitro, we adopted DP cell–activating culture (DPAC) medium, a previously established culture condition to induce DP properties in mesenchymal cells [31,32].

## 2. Results

### 2.1. Correlation between Vascular Pericyte and Preadipocyte Marker Expression with Treatment Outcome

In our previous study which assessed the therapeutic efficacy of autologous cell–based therapy using human DSC cells for male and female pattern hair loss, the total hair density and cumulative hair diameter at 3 × 10^5^ DSC cell–injected sites increased significantly compared with the placebo after 6 and 9 months [21]. Among DSC cell lines collected from 65 donors, 32 cell lines were randomly selected to evaluate the correlation between the DSC marker expression profile and treatment outcomes as demonstrated by the change in the measured total hair density compared with the placebo or 0 day as the baseline.

Selected DSC cell lines were cultured to examine the expression of representative vascular pericyte markers PDGFRβ, CSPG4, and nestin [27,28,29]. Subsequent correlation analysis was conducted to evaluate the correlation between their expression profiles and the most statistically significant treatment outcomes of total hair density at 6 and 9 months after DSC transplantation in the previous clinical study [21].

**Figure 1:**
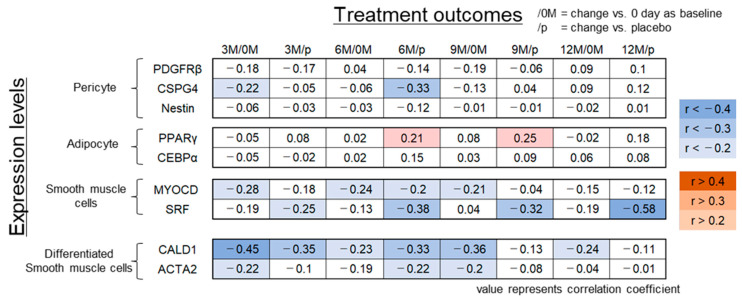
Correlation analysis between treatment outcome scores at each time point during the clinical study using DSC cell lines [21] and expression levels of differentiation markers. The results of correlation analysis between the treatment outcome score at 3, 6, 9, and 12 months vs. that at 0 day (3 M/0 M, 6 M/0 M, 9 M/0 M, and 12 M/0 M) or vs. the placebo (3 M/p, 6 M/p, 9 M/p, and 12M /p) and the expression levels of the indicated differentiation markers are shown. Each value represents the correlation coefficient. Red: positive correlation; blue: inverse correlation.

The CSPG4 expression level was inversely correlated with the treatment outcome at 6 months to a moderate extent (r = −0.33, *p* = 0.07). However, no correlation was found between PDGFRβ and nestin expression levels and treatment outcomes at any time point (Figure 1).

Next, the expression levels of preadipocyte markers PPARγ and CEBPα [30] were examined for their possible correlation with treatment outcomes. Weakly positive correlations were observed between PPARγ expression levels and treatment outcomes at 6 and 9 months after treatment (r = 0.21, 0.25, *p* = 0.27, 0.18) (Figure 1). However, such a correlation was not observed for CEBPα.

### 2.2. Correlation between Smooth Muscle Cell Marker Expression and Treatment Outcome

MYOCD and SRF are representative upstream genes that regulate the differentiation of mesenchymal cells into smooth muscle cells [25,26]. Expression levels of these markers in all DSC cell lines were measured and assessed for their correlation with treatment outcomes. MYOCD expression levels correlated negatively with treatment outcomes evaluated by comparison between pre- and post-treatment (day 0 and 6 or 9 months; r = −0.24, −0.21; *p* = 0.19, 0.25) and vs. the placebo at 6 months after DSC transplantation (r = −0.2, *p* = 0.27) (Figure 1). SRF expression levels consistently demonstrated an inverse correlation with the treatment outcome (vs. placebo at 3, 6, 9, and 12 months; r = −0.25, −0.38, −0.32, and −0.58, *p* = 0.20, 0.04, 0.09, and 0.001) (Figure 1).

Major smooth muscle cell marker expression levels demonstrated consistent negative correlations with treatment outcomes, and therefore we assessed additional smooth muscle cell markers CALD1 and ACTA2 [33]. CALD1 expression levels correlated negatively with treatment outcomes assessed by comparison between pre- and post-treatment (vs. day 0 at 3, 6, 9, and 12 months, r = −0.45, −0.23, −0.36, and −0.24, *p* = 0.02, 0.21, 0.05, and 0.20; vs. placebo at 3 and 6 months, r = −0.35 and −0.33, *p* = 0.06 and 0.07) (Figure 1). Additionally, ACTA2 expression levels and treatment outcomes correlated inversely (vs. 0 day at 9 months, r = −0.2, *p* = 0.29; vs. placebo at 6 months, r = −0.22, *p* = 0.24) (Figure 1).

### 2.3. DSC Cell Group with Distinct Therapeutic Efficacy Has Differential SRF and CALD1 Expression

To further investigate the utility of SRF and CALD1 as negative predictive markers for treatment outcomes, the DSC cell lines were divided into two groups—good responders and moderate/poor responders—by clinical improvement as calculated by the hair density vs. the placebo-treated group.

**Figure 2:**
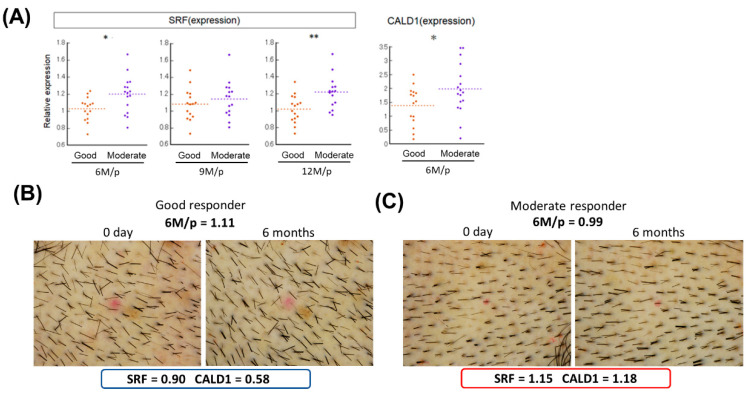
DSC cell lines derived from good responders express low levels of smooth muscle cell markers SRF and CALD1. (**A**) Relative expression levels of SRF and CALD1 in good and moderate/poor responder groups. Expression levels were normalized to GAPDH and the results are expressed as the mean ± S.D. Subdivision intoeach group was based on the treatment outcome score at indicated months vs. placebo at 6, 9, and 12 months (6 M/p, 9 M/p, and 12 M/p). Student’s *t*-test was applied for comparisons. * *p* < 0.05, ** *p* < 0.01. Pre- and post-treatment trichoscopic images of tested areas of a good (**B**) and moderate/poor (**C**) responder at 6 months are shown with the treatment outcome score (6 M/p) and values of relative expression levels of SRF and CALD1.

Intriguingly, SRF was downregulated in the good responder group compared with the moderate/poor group at both 6 and 12 months (*p* < 0.05 and *p* < 0.01) (Figure 2A). CALD1 showed an analogous expression pattern at 6 months (*p* < 0.05) (Figure 2A). Pre- and post-treatment trichoscopic images of tested areas of good and moderate responders at 6 months are presented in Figure 2B,C, respectively. The expression levels of SRF and CALD1 in DSC cells were relatively lower (SRF = 0.90, CALD1 = 0.58) in a good responder compared with a moderate responder (SRF = 1.15, CALD1 = 1.18) (Figure 2B,C).

These findings supported that DSC cell lines with potent therapeutic efficacy could be selected based on low expression levels of smooth muscle markers represented by SRF and CALD1.

### 2.4. In Vitro Differentiation Toof DP Cells Enhances Correlation with Treatment Outcome

**Figure 3:**
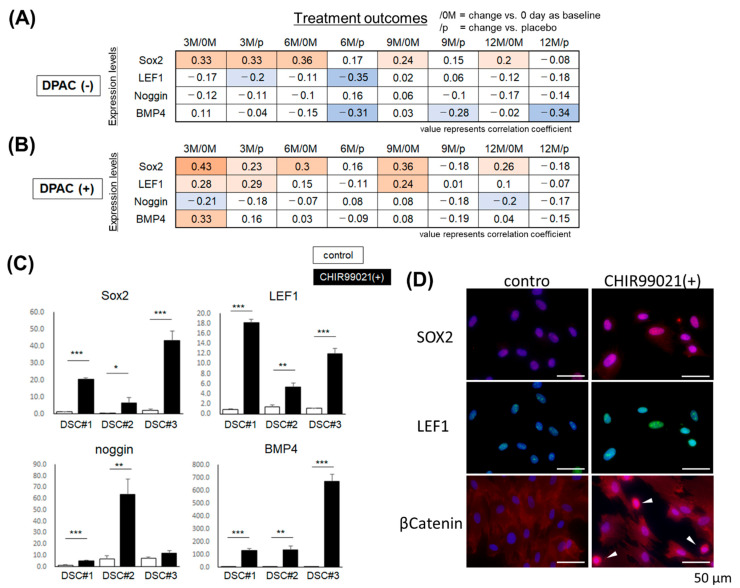
Effect of DPAC medium and CHIR99021 treatment on DSC cells. (**A**,**B**) Correlation between the treatment outcome score at 3, 6, 9, and 12 months vs. day 0 (3 M/0 M, 6 M/0 M, 9 M/0 M, and 12 M/0 M) or vs. placebo (3 M/p, 6 M/p, 9 M/p, and 12 M/p) during the clinical study using DSC cell lines [21], and the expression levels of DP markers. Each value represents the correlation coefficient. Red: positive correlation; blue: inverse correlation. (**C**) Relative expression levels of Sox2, LEF1, Noggin, and BMP4 in three DSC cell lines (DSC#1–#3) cultured in medium (control) or with 3 µM CHIR99021 (*n* = 3). Expression levels were normalized to GAPDH and the results are expressed as the mean ± S.D. Student’s *t*-test, * *p* < 0.05, ** *p* < 0.01, *** *p* < 0.001. (**D**) Immunostaining of DSC cells cultured in control or CHIR99021-containing medium for SOX2 (red), LEF1 (green), and β-catenin (red). Blue indicates Hoechst 33,342 nuclear staining. Arrowheads: β-catenin nuclear translocation. Bars = 50 µm.

On the basis of the hypothesis that injected DSC cells improved hair loss putatively by forming DPs in miniaturized hair follicles in the clinical study, in vitro differentiation of DSC cells toward DP cells may facilitate assessment of the therapeutic potential of DSC cells. Therefore, DP cell–activating culture (DPAC) medium, a previously established culture condition that induces DP properties in mesenchymal cells [31,32], was adopted to confer DP properties to DSC cells. The expression levels of DP biomarkers SOX2, LEF1, Noggin, and BMP4 [32,34], which reflect hair-inductive capacity, were evaluated in DSC cells before and after DPAC treatment (Figure 3A,B).

The SOX2 and LEF1 expression levels in the DSC cells before DPAC induction demonstrated moderate positive and inverse correlations, respectively, with the treatment outcome (Figure 3A,B). Intriguingly, SOX2 expression levels in the DSC cells after the DPAC treatment more consistently demonstrated a positive correlation with the treatment outcome (vs. day 0, at 3, 6, 9, and 12 months; r = 0.43, 0.3, 0.36, and 0.26, *p* = 0.02, 0.09, 0.04, and 0.15) (Figure 3A,B). Additionally, LEF1 expression levels were influenced by DPAC treatment and exhibited a positive correlation with the treatment outcome (vs. day 0, at 3 and 9 months, r = 0.28 and 0.24, *p* = 0.12 and 0.18; vs. placebo, at 3 months, r = 0.29) (Figure 3B). Similarly, the correlation between BMP4 expression levels and the treatment outcome was reversed from negative to positive by DPAC treatment (Figure 3A,B).

These findings suggested that DPAC treatment modulated DSC properties to those resembling DPs and potentially augmented the magnitude of the correlation between putative predictive marker expression and treatment outcome.

DPAC medium contains recombinant BMP2, basic FGF, and WNT activator; 6-bromoindirubin-3′-oxime (BIO) as a GSK3β inhibitor. Both LEF1 and BMP4 are downstream target genes of WNT signaling [35,36], implying that WNT stimulation alone would be sufficient for modulation of a less detectable correlation between possible marker expression and treatment outcomes. Such simplification should be beneficial for clinical application.

CHIR99021 is a potent WNT activator as a GSK3β inhibitor [37] and increases the hair follicle generation ability of DP cells [19]. The addition of CHIR99021 to DSC cell culture resulted in significant upregulation of SOX2, LEF1, Noggin, and BMP4 analogously to DPAC treatment except for Noggin in one DSC line (Figure 3C). Immunostaining confirmed upregulation of SOX2 and LEF1 that were localized to nuclei in CHIR99021-treated DSC cells (Figure 3D). An increase in the translocation of β-catenin to nuclei was also observed in CHIR99021-treated DSC cells (Figure 3D).

These data implied that CHIR99021 alone could substitute for DPAC to reveal an otherwise less detectable correlation between predictive marker expression and treatment outcomes.

### 2.5. DSC Cells with a High Hair Generative Ability Express Smooth Muscle Cell–Related Genes at Low Levels

**Figure 4:**
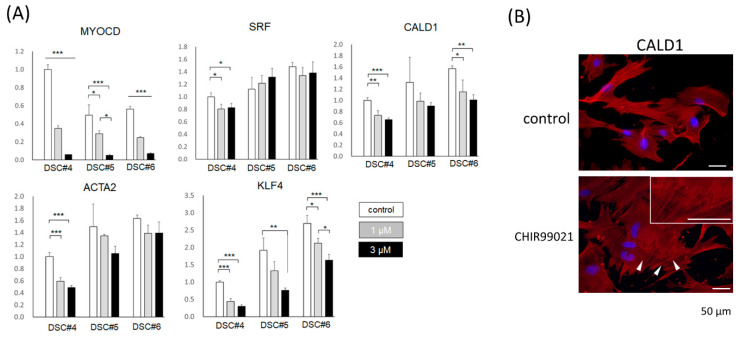
CHIR99021 treatment suppresses the expression of smooth muscle–related genes in DSC cells. (**A**) Relative expression levels of MYOCD, SRF, CALD1, ACTA2, and KLF4 in three DSC cell lines (DSC#4–#6) treated with the control and 1 or 3 µM CHIR99021 (*n* = 3). Expression levels were normalized to GAPDH, and the results are expressed as the mean ± S.D. Tukey–Kramer test, * *p* < 0.05, ** *p* < 0.01, *** *p* < 0.001. (**B**) Immunostaining of DSC cells treated with control or CHIR99021(+) medium for CALD1 (red). Blue; Hoechst 33,342 nuclear staining. Arrowheads: stress fibers associated with CALD1 (red). Bars = 50 μm.

To assess whether DSC cells upregulate expression of smooth muscle cell markers, including SRF and CALD1, in response to CHIR99021, three DSC cell lines were treated with or without CHIR99021. Significant downregulation of MYOCD, an upstream gene that regulates differentiation into smooth muscle cells [26] was observed in a dose-dependent manner (*p* < 0.05) (Figure 4A). SRF was downregulated in one DSC line compared with the control (*p* < 0.05). However, the trend was inconsistent in the other two lines examined (Figure 4A). CALD1 and ACTA2 expression tended to decrease in statistical significance in two (*p* < 0.05) and one (*p* < 0.001) DSC cell lines examined, respectively (Figure 4A). In line with this, immunostaining revealed a decrease in CALD1 expression in CHIR99021-treated DSC cells compared with the untreated control (Figure 4B), and stress fibers were detected in the tested cells, which has been reported in DP cells (Figure 4B). KLF4 suppresses ACTA2 expression and inhibits differentiation to smooth muscle cells induced by MYOCD [38]. Unexpectedly, CHIR99021 treatment suppressed KLF4 expression dose-dependently in all DSC lines (*p* < 0.05).

These findings suggested the advantage of adopting CHIR99021 for preclinical in vitro evaluation of DSC cell bioactivity to improve hair loss by intensifying the downregulation of smooth muscle cell marker gene expression to putatively enable easier detection of correlations with the treatment outcome.

## 3. Discussion

The hair-inductive capacity of DSC cells has long been recognized and endorsed by a recent transplantation study as mentioned above. However, the therapeutic effect was variable among the recipients. On the basis of an estimation taking into account the number of DSC cells used for this clinical study, the number of DSC cells necessary for treating male and female pattern hair loss in a real-world setting would be enormous, resulting in high medical expenses. The physical burden can also be minimized by selecting highly hair-inductive DSC cells for treatment. Thus, it is pivotal to establish a methodology to predict the therapeutic efficacy of prepared DSC cells at an early stage of primary culture.

As a precedent, mesenchymal stem cell transplantation has been used to treat ischemic and inflammatory immune diseases with favorable outcomes [39]. Attempts have been made to selectively obtain optimal cell populations for individual therapeutic goals by monitoring marker gene expression as an index, such as TWIST1 [39]. DSC cells derived from manually microdissected tissues are likely to be more heterogenous than mesenchymal stem cells, which may lead to difficulty in identifying such predictive markers. However, such an approach may be reasonable and was adopted in this study.

Pericyte and smooth muscle cell markers were investigated as candidates for therapeutic outcome indicators. DS cells have been reported to serve as a source of myofibroblasts with smooth muscle cell properties [22,23]. Both DS cells and pericytes also give rise to myofibroblasts [24]. Myofibroblasts and pericytes emerge at the time of wound healing and are responsible for tissue repair either as cellular material or by secreting repair factors [23,40]. Because de novo hair follicle induction occurs simultaneously in the healing of large mouse wounds, the transdifferentiation potential of DS cells for pericytes or smooth muscle cells may correlate with the therapeutic potential of DSC cells. Differentiation marker expression of adipocytes has been similarly examined because human DS cells can differentiate into adipocytes and osteocytes [41], and adipocyte lineage cells have been implicated in contributing to hair regeneration [42]. Although this approach allowed identification of several markers whose expression levels were negatively correlated with therapeutic outcomes, adaptation of high-throughput analysis platforms, including microarray and RNA sequencing, may enable the detection of other markers, which is an important next step.

In contrast to our expectations, no positively correlated markers were found, at least among those examined. This is probably because the markers studied were associated with differentiation into respective cell lineages. Theoretically, the DSC cell capacity to replenish DS—or, more importantly, DP—needs to be highly plastic. Some DS cells, which are considered to be DP progenitors, have been suggested to possess stem cell properties [20]. Skin-derived precursor cells, a multipotent cell population capable of differentiating into multiple mesodermal lineages including smooth muscle cells and adipocytes, have been suggested to originate from Sox2-positive DP cells and DSC cells [43]. Intriguingly, the Sox2 expression level in DSC cells, which was increased by differentiation into DP cells, correlated positively with treatment outcome at several time points, indicating that less-committed DSC cells with stem cell characteristics might be optimal for ttransplantation to improve therapeutic efficacy. At least, on the basis of the findings obtained in this study, pericyte and adipocyte characteristics are less likely to be involved in the mechanism underlying DSC therapeutic potential.

In the current study, DSC cell lines were randomly selected for correlation analyses, which was a major limitation. A marker with higher correlation may have been found if DSC cell lines were selected from patients with larger differences in treatment outcomes. Most markers exhibited an inverse correlation at 6 months irrespective of the magnitude. The findings are reasonable because clinical outcomes were remarkable at this time. Conversely, smooth muscle cell–related marker expression consistently demonstrated negative correlations during the time course, which further supported the utility of assessing the expression levels of these markers.

DP cells lose their intrinsic properties during cell culture, suggesting the analogous impairment of bioactivity in DSC cells in vitro. This may hamper the identification of marker candidates. Importantly, treatment with DPAC medium—and more simply, CHIR99021 alone—enhanced the changes in putative marker expression. This finding suggests that the addition of a WNT agonist would be an effective approach to improve the sensitivity of therapeutic efficacy detection. Because Wnt signaling improves the hair follicle–inducing and regenerative abilities of DS cells [44,45], treatment of DSC cells with a WNT agonist would also ameliorate the therapeutic potential of treated cells and may therefore provide a strategy to further improve treatment outcome. Given that smooth muscle cell–related marker expression consistently demonstrated negative correlations, a treatment to lose the property of smooth muscle cells possibly may enhance the therapeutic potential of DSC cells, which can lead to inverse upregulation of SOX2, LEF1, Noggin, and BMP4.

This study had several limitations. The sample size was small and the treatment outcomes were obtained from a clinical study that was mainly designed as a proof-of-concept study to evaluate the safety and efficacy of DSC cell transplantation. Results obtained in further clinical studies to verify the improvement in therapy would be more appropriate for identification of predictive markers. Additionally, methodologies other than gene expression analysis need to be adopted to confirm the results. DSC cell lines derived from good responders express lower levels of SRF and CALD1 at transcription levels compared with moderate responders (Figure 2). However, the difference may be relatively small to be adopted as biomarkers. In the current study, additional analyses, including Western blot to analyze protein expression level, were not feasible because of the shortage of the remaining samples left behind in the clinical study [21]. Validation of the usefulness of SRF and CALD1 as biomarkers at the protein level should represent an important next step to enhance the significance of the findings observed in this study.

Although further investigation is needed to prove more convincingly the utility of the identified markers, our findings suggest that a pre-transplantation assessment of the therapeutic potential of DSC cells for male and female pattern hair loss is possible. Further accumulation of clinical data and DSC samples combined with high-throughput analyses should facilitate more robust identification of predictive markers.

## 4. Materials and Methods

### 4.1. Scoring of the Treatment Outcome

Among DSC cell lines collected from 65 donors in clinical studies [21], 32 cell lines were randomly selected for the present study to evaluate the correlation between the DSC marker expression profile and treatment outcomes. All subjects signed informed consent forms approved by the Institutional Review Board at Tokyo Medical University and the Toho University Ohashi Medical Center and the Certified Committee for Regenerative Medicine at the Tokyo Medical University under the Act on the Safety of Regenerative Medicine (approval no. 2016001). The hairs at the DSC cells injection sites of each subject were clipped to 1 mm length before the injection and at 3, 6, 9, and 12 months later, and Phototrichogram images were taken with a digital camera. The total hair density (hairs/cm^2^) was measured using image analysis system software (Hybrid Measure, Inotech Corp, Hiroshima, Japan) as previously described [21]. The measured total hair densities of the recipients of the 32 DSC cell lines [21] were used to calculate the treatment outcome score. The measured total hair densities at 3, 6, 9, and 12 months were divided by those of the corresponding placebo control at each time point or by one at 0 day as baseline. The change vs. the placebo or day 0 were used as the treatment outcome score. To assess the correlation between the treatment outcome score and the expression level of examined genes, Pearson correlation analysis was used to calculate the correlation coefficient with the R program.

The selected 32 DSC cell line recipients were subdivided into the top half and the other half in order of treatment outcome score as good and moderate/poor responder groups, respectively. Subdivided groups by the score at 6 months vs. the placebo are analyzed in the Supplementary Figure S1, and subdivided groups by the score at 6, 9, and 12 months vs. the placebo are presented in Figure 2.

### 4.2. Cell Culture and cDNA Library Preparation

The DSC cells remaining after completion of the clinical usage were used for the present study with one to three additional passages. Seeded DSC cells were cultured in Dulbecco’s modified Eagle’s medium (Life Technologies, Carlsbad, CA, USA) supplemented with 10% fetal bovine serum and 1% antibiotic/antimycotic (100×) (Nacalai Tesque, Kyoto, Japan), and maintained in a 37 °C incubator with 5% CO_2_. At 2 days after seeding, the total RNA was extracted with an RNeasy Mini QIAcube Kit (Qiagen) (Hilden, Germany) or a SV 96 Total RNA Isolation System in accordance with the manufacturer’s protocols. For DPAC, DSC cells were cultured in DPAC medium for an additional 3 days in accordance with previous reports [31,32].

To assess the effect of CHIR99021, newly isolated DSC cells were prepared (DSC#1–#4). DSC#5 and #6 were the DSC cell lines used in the clinical study. Human scalp tissues were obtained from a plastic surgeon with written informed consent and with approval from the Ethics Committee of Shiseido. The study was conducted in accordance with the Declaration of Helsinki. Human DSC cells were expanded from explant cultures in MesenPRO RS medium (Life Technologies). Seeded DSC cells were maintained in a 37 °C incubator with 5% CO_2_. CHIR99021 was purchased from Axon Medchem (Groningen, Netherlands). For CHIR99021 stimulation, DSC cells were cultured in a medium containing CHIR99021 (3 µM) for 3 days, followed by total RNA extraction.

cDNA was synthesized using a high-capacity RNA-to-cDNA kit (Applied Biosystems, Carlsbad, CA, USA) or a SuperScript^®^ III First-Strand Synthesis System (Thermo Fisher Scientific, Waltham, MA, USA) in accordance with the manufacturers’ protocols.

### 4.3. Quantitative Real-Time Reverse-Transcription PCR (qRT-PCR)

Gene expression was investigated using a LightCycler 480 SYBR Green Master (Roche, Basel, Switzerland) or a SsoAdvanced™ Universal SYBR^®^ Green Supermix (Bio-Rad, Hercules, CA, USA) in accordance with the manufacturer’s protocols (40 cycles, 60 °C as the primer annealing temperature). Quantification of expression was performed using the 2^−ΔΔCT^ method [46]. Relative expression levels were calculated using glyceraldehyde 3-phosphate dehydrogenase (GAPDH) as a reference gene. Primer pairs are presented in the Supplementary Table S1. Primer pairs were verified by analyzing the melting curve with single amplicons. Primer pairs for LEF1, BMP4, Noggin, and KLF4 were purchased from Qiagen as QuantiTech primers.

### 4.4. Immunostaining

For the immunocytochemistry of DSC cells, SOX2, LEF1, β-catenin, and CALD1- were detected with 100- to 500-fold diluted antibodies (anti-SOX2 antibody, MAB2018; R&D Systems, Minneapolis, MN, anti-LEF1 antibody, PA5-82000; Invitrogen, Carlsbad, CA, USA, anti-β-catenin antibody, 610153; BD Pharmingen, San Diego, CA, USA, anti-CALD1 antibody, 66693-1-Ig; Proteintech, Rosemont, IL, USA) and Alexa 488- or 594-conjugated secondary antibodies (Thermo Fisher Scientific). After DSC cells in a cell chamber slide were fixed with 4% paraformaldehyde for 15 min, immunostaining was performed as described previously [47]. After permeabilization with 5% Triton X-100/PBS for 10 min, DSC cells were immunostained with primary antibodies and Alexa 488- or 594-conjugated as secondary antibodies. Stained DSC cells were mounted with Vectashield mounting medium (Vector Laboratories, Burlingame, CA, USA) and examined under a fluorescence microscope.

## Data Availability

Not applicable.

## Supplementary Materials

The following supporting information can be downloaded at: https://www.mdpi.com/article/10.3390/ijms23137125/s1.
