# Supplementary material for: Changes in the Expression of Smooth Muscle Cell–Related Genes in Human Dermal Sheath Cup Cells Associated with the Treatment Outcome of Autologous Cell–Based Therapy for Male and Female Pattern Hair Loss
Source: Int J Mol Sci. 2022 Jun 27;23(13):7125. doi: 10.3390/ijms23137125 (PMC9266963; doi:10.3390/ijms23137125)
Supplement: Supplementary file 1 [file ijms-23-07125-s001.zip › ijms-1764208-supplementary.pdf]

Supplementary Materials

	3M/0M	3M/p	6M/0M	6M/p	9M/0M	9M/p	12M/0M	12M/p
Good responder	1.05 ± 0.07 **	1.04 ± 0.08 **	1.03 ± 0.08 *	1.05 ± 0.04 ***	1.018 ± 0.058 *	1.04 ± 0.06 *	1.02 ± 0.08	1.03 ± 0.06 **
Moderate/poor responder	0.99 ± 0.04	0.97 ± 0.05	0.97 ± 0.06	0.95 ± 0.04	0.97 ± 0.06	0.99 ± 0.06	0.98 ± 0.05	0.98 ± 0.05

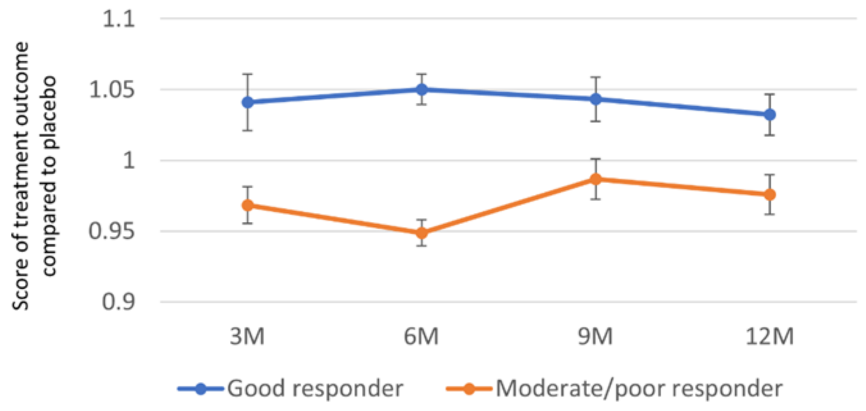

Figure S1. Classification of patient groups by the treatment outcome. Patients who participated in the clinical study adopting DCS cell injection into androgenetic alopecia lesions were divided into good responder (n = 16) and moderate/poor responder (n = 16) groups based on the treatment outcome at 6 months vs. the placebo calculated by the change in measured total hair density. The score of each group at 3, 6, 9, and 12 months vs. day 0 (3M/0M, 6M/0M, 9M/0M, and 12M/0M), and vs. the placebo (3M/p, 6M/p, 9M/p, and 12M/p) was plotted. Scores are expressed as the mean ± S.D. Student's t-test was applied for comparisons between the two groups (*P < 0.05, **P < 0.01, ***P < 0.001). The line graph illustrates the data of 3, 6, 9, and 12 months vs. the placebo (mean ± S.E.).

Table S1. Primer pairs used for qRT-PCR.

Gene name	**Forward (5′ to 3′)**	**Reverse (5′ to 3′)**
ACTA2	ATTGCCGACCGAATGCAGAAGG	GGCTTCATCGTATTCCTGTTTGCTG
CALD1	GTCTGTGCAGAAAAGCAGTGG	TGCCGGCTTTGTAGGTTTTG
MYOCD	CCCAGCCACCAGTCAGATG	GGCTTTTTGTGGCGGTTCTT
SRF	CCTACCAGGTGTCGGAGTCT	AGGTGCTAGGTGCTGTTTGG
PPARγ	CTTCACTACTGTTGACTTCTCCAGC	GAGGCTTATTGTAGAGCTGAGTCTTC
CEBPα	AAGTCGGTGGACAAGAACAGCAAC	AGTTCGCGGCTCAGCTGTTC
PDGFRβ	CTGCTTCTCGAGAGACTGTTGG	TGCACGGCAGTATAGAGGACG
CSPG4	CATGATACCCTAGAGCTCCAGC	CCAAGAGATTGGAGGCATCCAG
nestin	TCAAGATGTCCCTCAGCCTGG	ACTGGGAGCAAAGATCCAAGACG
Sox2	GGAAATGGGAGGGGTGCAAAAGAGG	TTGCGTGAGTGTGGATGGGATTGGTG
GAPDH	GAGTCAACGGATTTGGTCGT	TGGGATTTCCATTGATGACA
